# γδ T Cells Provide Protective Function in Highly Pathogenic Avian H5N1 Influenza A Virus Infection

**DOI:** 10.3389/fimmu.2018.02812

**Published:** 2018-12-04

**Authors:** Peng Dong, Xiangwu Ju, Yiwu Yan, Siya Zhang, Menghua Cai, Huaishan Wang, Hui Chen, Yu Hu, Lianxian Cui, Jianmin Zhang, Wei He

**Affiliations:** ^1^State Key Laboratory of Medical Molecular Biology, Department of Immunology, Research Center on Pediatric Development and Diseases, Institute of Basic Medical Sciences, Chinese Academy of Medical Sciences and School of Basic Medicine, Peking Union Medical College, Beijing, China; ^2^Department of Rheumatology and Immunology, Beijing Jishuitan Hospital, 4th Medical College of Peking University, Beijing, China; ^3^State Key Laboratory of Medical Molecular Biology, Department of Biochemistry and Molecular Biology, Institute of Basic Medical Sciences, Chinese Academy of Medical Sciences and School of Basic Medicine, Peking Union Medical College, Beijing, China

**Keywords:** γδ T cells, highly pathogenic avian H5N1 influenza A virus, hemagglutinin, sialic acid receptors, phosphatase calcineurin inhibitor

## Abstract

Given the high mortality rate (>50%) and potential danger of intrapersonal transmission, highly pathogenic avian influenza (HPAI) H5N1 epidemics still pose a significant threat to humans. γδ T cells, which participate on the front line of the host immune defense, demonstrate both innate, and adaptive characteristics in their immune response and have potent antiviral activity against various viruses. However, the roles of γδ T cells in HPAI H5N1 viral infection remain unclear. In this study, we found that γδ T cells provided a crucial protective function in the defense against HPAI H5N1 viral infection. HPAI H5N1 viruses could directly activate γδ T cells, leading to enhanced CD69 expression and IFN-γ secretion. Importantly, we found that the trimer but not the monomer of HPAI H5N1 virus hemagglutinin (HA) proteins could directly activate γδ T cells. HA-induced γδ T cell activation was dependent on both sialic acid receptors and HA glycosylation, and this activation could be inhibited by the phosphatase calcineurin inhibitor cyclosporin A but not by the phosphatidylinositol 3-kinase (PI3-K) inhibitors wortmannin and LY294002. Our findings provide a further understanding the mechanism underlying γδ T cell-mediated innate and adoptive immune responses against HPAI H5N1 viral infection, which helps to develop novel therapeutic strategies for the treatment of H5N1 infection in the future.

## Introduction

The highly pathogenic avian H5N1 influenza A virus poses a high risk for a global pandemic (1–4). To date, more than 800 people worldwide have been infected, and the mortality is over 50% (5). Most human cases of H5N1 infection present as progressive pneumonia, and the ultimate cause of death is the most severe form of acute lung injury, which is called acute respiratory distress syndrome (ARDS) (6, 7). Many viruses, including H5N1, are mutating and producing strains that are resistant to currently known vaccines or antiviral drugs (8, 9). Thus, it is imperative to develop alternative therapeutic strategies.

Unlike αβ T cells, γδ T cells demonstrate both innate and adaptive characteristics in their immune response and have potent antiviral activity against different viruses, including influenza A virus (10, 11), human immunodeficiency virus (HIV) (12), cytomegalovirus (CMV) (13), and herpes simplex virus 1 (HSV-1) (14). Recent studies demonstrated that γδ T cells expanded by the phosphoantigen isopentenyl pyrophosphate (IPP) were able to kill monocyte-derived macrophages infected by human (H1N1) and avian (H9N2 or H5N1) influenza viruses (15, 16). Peripheral γδ T cells can be rapidly activated by influenza virus (H1N1, H2N2, and H3N2) infection via a mechanism related to the mevalonate pathway (17). However, the interaction between human γδ T cells and highly pathogenic avian influenza (HPAI) H5N1 viruses remains poorly understood. After H5N1 infection, numerous influenza virus particles are released from the infected epithelia or macrophages (18). In the infection microenvironment, γδ T cells undoubtedly encounter these virus particles. Therefore, investigating the direct interaction of γδ T cells with HPAI H5N1 viruses is important. However, no reports of the direct effects of HPAI H5N1 viruses on human γδ T cells are yet available.

In this study, we investigated the roles of γδ T cells in the defense against HPAI H5N1 viral infection. We found that γδ T cells provided crucial protective function during HPAI H5N1 viral infection. HPAI H5N1 viruses could directly activate γδ T cells via natural HA trimers, initiating γδ T cell-mediated innate and adoptive immune response. Our findings provide a further understanding the mechanism underlying γδ T cell-mediated innate and adoptive immune responses against HPAI H5N1 viral infection.

## Materials and Methods

### Influenza Viruses and Cells

The influenza viruses used in this study were an H5N1 [A/Jilin/9/2004 (H5N1)] and an influenza A virus [A/new/Caledonia/20/1999 (H1N1)]. Live virus experiments were performed in biosafety level 3 facilities under governmental and institutional guidelines. Viruses were propagated by inoculation into 10- to 11-day-old specific pathogen-free (SPF) embryonated fowl eggs via the allantoic route. Hemagglutinating allantoic fluid (AF) was collected from eggs and used directly (19). All animal experiments were conducted with the approval of the Institutional Animal Care and Use Committee of the Institute of Military Veterinary Medicine, Academy of Military Medical Sciences. MDCK cells were purchased from the Peking Union Medical College Cell Culture Center and cultured in DMEM (Gibco Invitrogen, USA) medium supplemented with 10% FBS, 100 U/ml penicillin and 100 μg/ml streptomycin at 37°C under a 5% carbon dioxide atmosphere. The human lung adenocarcinoma A549 cell line was purchased from the American Type Culture Collection (ATCC) and cultured in F-12/Ham (Gibco Invitrogen, USA) medium supplemented with 10% FBS, 100 U/ml penicillin and 100 μg/ml streptomycin at 37°C under a 5% carbon dioxide atmosphere.

### Expansion and Isolation of γδ T Cells

γδ T cells were expanded from human peripheral blood monocytes (PBMCs) as described previously (20). Briefly, fresh PBMCs separated from the peripheral blood of healthy donors by Ficoll-Hypaque (Pharmacia, Sweden) density gradient centrifugation were grown in RPMI-1640 medium (Gibco BRL, USA) supplemented with 10% FCS and IL-2 (200 UI/ml) in 24-well culture plates coated with an immobilized anti-pan-TCR γδ mAb (0.05 mg/ml, Immunotech, France). After 10 to 12 days of culture, the purity of the γδ T cells was more than 85%, as assessed by flow cytometric analysis. γδ T cells were further purified and concentrated to a purity of up to 98% using the TCRγ/δ+ T Cell Isolation Kit (Miltenyi Biotec, Germany) in accordance with the manufacturer's instructions.

### Viral Titration

Tenfold dilutions of the virus were used to inoculate MDCK cells in a 96-well plate, and infected cells were maintained in culture for 72 h. Viral titers were calculated using the Reed-Muench method and are expressed as the tissue culture infectious dose (TCID50) per milliliter of supernatant.

### Animal Handling

The animal experiments were conducted in the animal facility of the Institute of Basic Medical Sciences, Peking Union Medical College and the Institute of Military Veterinary Medicine, Academy of Military Medical Sciences, in accordance with governmental and institutional guidelines. Wild-type (WT) C57BL/6 mice were purchased from Vital River, a Charles River company in China. TCR-δ^−/−^ mice were bred from B6.129P2-*Tcrb*^*tm*1*Mom*^/*Tcrd*^*tm*1*Mom*^ mice provided by professor Mingzhao Zhu (Key Laboratory of Infection and Immunity, Institute of Biophysics, Chinese Academy of Sciences). All mice were housed in an SPF facility. Lung injury was induced via the intratracheal instillation of AF vehicle or virus as previously reported (21). Briefly, WT and TCR-δ^−/−^ mice were anesthetized by sodium pentobarbital and inoculated intranasally with 0.8 × 10^5^ TCID_50_ H5N1 virus. At 4 days post infection (DPI), mice were killed, and the lungs of each group of three mice were fixed in formalin and were then embedded in paraffin. Sections of 6 μm thickness were obtained and stained with hematoxylin-eosin. At 4 DPI, the wet weight of the lungs of three mice was measured. The lungs were then heated to 68°C for 24 h, and the dry weight of the lungs was recorded; the wet/dry ratios were then calculated. The survival percentages and body weights in each group of 10 mice were monitored daily for 14 days. Survival data were analyzed by Kaplan-Meier survival analysis using GraphPad Prism 5 software.

### Expression of Recombinant HA (rHA) Proteins

Monomeric and trimeric rHA proteins were expressed and purified using a baculovirus-insect cell system (Invitrogen, Thermo Fisher scientific, USA) as described previously (22). First, the HA ectodomain DNA fragment of A/Anhui/1/2005 (H5N1, Accession No. DQ371928) and His tag were cloned into the transfer vector PacGP67b (BD Biosciences Pharmingen, USA) to allow the efficient secretion of monomeric rHA proteins. A new construct containing the bacteriophage T4 fibritin fold on trimerization sequence was generated to allow the efficient secretion of trimeric rHA proteins as previously reported (23). Next, Sf9 cells were cotransfected with the monomeric or trimeric rHA transfer vectors and linearized baculovirus DNA (Invitrogen, Thermo Fisher scientific, USA) to produce recombinant baculoviruses containing the HA genes. Transfection and virus amplification were carried out according to the baculovirus expression system manual. The supernatant from infected Sf9 cells was collected and purified by Ni-NTA chromatography (GE Healthcare, USA) against the C-terminal His tag. Western blotting was performed using anti-His or anti-HA antibodies to confirm the rHA proteins. To demonstrate that the expressed HA fragments were properly folded, they were analyzed by a Viscotek 270 Max GPC/SEC system according to the manufacturer's instructions (Malvern, UK). Gel filtration chromatography was conducted using P4000 and P2500 columns (Malvern, UK) with a running buffer (pH 8.0) composed of 135 mm NaCl, 135 mm KCl, 1.5 mm KH2PO4, and 1.0 mm Na2HPO4·12H2O.

### Hemagglutination Assay

Human erythrocytes were separated from whole blood of healthy donors. After isolation and washing, 50 μl of a 0.75% human red blood cell (RBC) suspension was added to 50 μl volumes of 2-fold serial dilutions of purified rHA proteins in a U-bottom 96-well plate (BD Falcon, USA; total volume, 100 μl). Agglutination was read after incubation for 60 min at room temperature. As a control, phosphate-buffered saline (PBS) was used instead of rHA.

### Flow Cytometric Analysis

Freshly isolated γδ T cells were resuspended in PBS containing 1% bovine serum albumin. The cells were then incubated with PE-conjugated anti-CD69 (BioLegend, USA) or isotype control antibodies for 20 min at 4°C. After being washed with PBS for three times, the cells were analyzed on an Accuri C6 flow cytometer (BD Biosciences, USA). The data are presented as either the percent positive cells or the mean fluorescence intensity.

### IFN-γ Secretion Assay

A total of 1 × 10^6^ γδ T cells per well were seeded into 48-well plates and were then treated with PBS, purified rHA proteins for 12, 24, and 48 h. Cell-free supernatants were collected, and IFN-γ secretion was detected with the human IFN-γ Immunoassay Kit (R&D Systems, USA) according to the manufacturer's instructions.

### Ca^2+^ Video Imaging

Measurement of the intracellular Ca^2+^ levels was performed in γδ T cells loaded with 2.5 μm fluo-4 AM (Invitrogen, USA) for 45 min at room temperature in HBSS. γδ T cells were washed, resuspended in HBSS and seeded on Lab-Tek^TM^ glass chamber slides (Nunc, Naperville, IL, USA) for 1 h at room temperature. When indicated, HA-his (3 μg/ml), HAF (3 μg/ml) and an anti-CD3 mAb (5 μg/ml) were added (time point: 0 min). Measurements of the intracellular Ca^2+^ responses were performed with an UltraVIEW VoX 3D Live Cell Imaging System (PerkinElmer, USA). γδ T cells were illuminated every 20 s and monitored for changes in intracellular Ca^2+^ levels by videomicroscopy for 1 h.

### Lectin Staining

To determine the surface expression level of sialic acid receptors, γδ T cells were incubated with fluorescein-labeled *Sambucus nigra* lectin (SNA, Vector, USA; 10 μg/ml; specific for SAα2,6Gal) and fluorescein-labeled *Maackia amurensis* lectin I (MAL I, Vector, USA; 10 μg/ml; specific for SAα2,3Gal) for 30 min at 4°C in the dark. γδ T cells were washed, resuspended in PBS and seeded on Lab-Tek^TM^ glass chamber slides (Nunc, Naperville, IL, USA) for 1 h at room temperature. The samples were analyzed using a laser scanning confocal microscope (Olympus, Japan).

### Statistical Analyses

All data are presented as the means ± SEMs. Measurements at single time points were analyzed using ANOVA, and if these measurements demonstrated significance, they were further analyzed by a two-tailed *t*-test. Survival data were subjected to Kaplan-Meier survival analysis. All statistical tests were performed with GraphPad Prism 5.0 (GraphPad Software, San Diego, CA, USA). *P* < 0.05 indicates statistical significance.

## Results

### Deficiency of γδ T Cells Exacerbates H5N1 Virus-Induced Lung Injury

**Figure 1:**
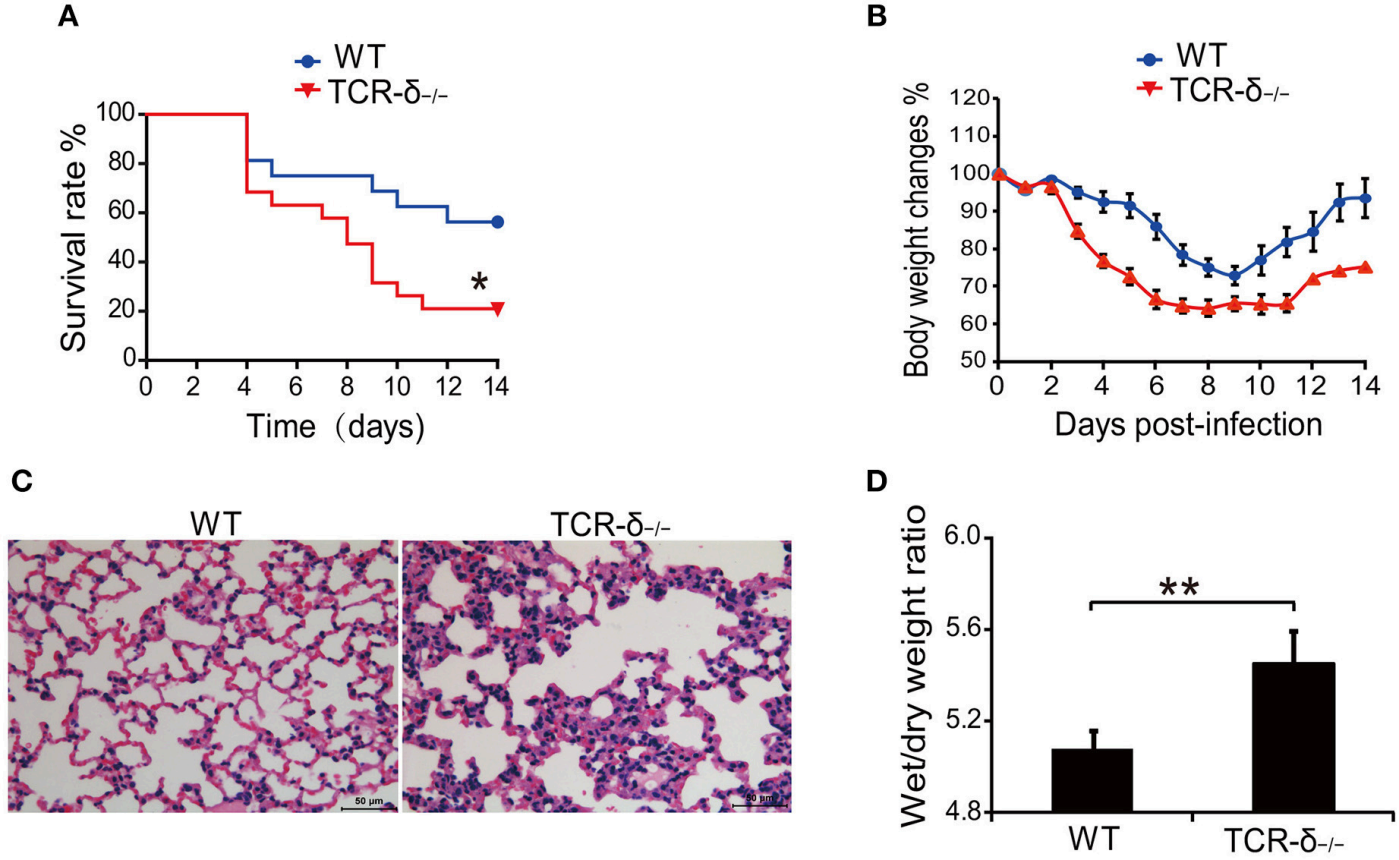
TCR-δ^−/−^ mice exacerbate H5N1 virus-induced lung injury. WT and TCR-δ^−/−^ mice were intranasally inoculated with 0.8 × 10^5^ TCID_50_ H5N1 virus. **(A)** Survival curves of WT (*n* = 10) and TCR-δ^−/−^ (*n* = 10) mice. **(B)** Changes in body weight. Values are the means ± SEMs from 10 mice. **(C)** Representative pathological images from the lungs of WT and TCR-δ^−/−^ mice at 4 DPI. **(D)** Wet/dry ratios of lung tissues at 4 DPI. Values are the means ± SEMs from six mice. ^*^P < 0.05, ^**^P < 0.01, Student's *t-*test, compared with WT mice.

To investigate the role of γδ T cells in H5N1 virus-induced lung injury, we examined the effect of γδ T cell deficiency on the host response to H5N1 avian flu virus infection. The results showed that H5N1 virus-infected TCR-δ^−/−^ mice died significantly earlier than did the WT controls (Figure 1A). Compared with the WT controls, the TCR-δ^−/−^mice experienced a greater loss of body weight (Figure 1B). The pathological alterations observed in the stained mouse lung tissues were also more severe in the TCR-δ^−/−^mice than in the WT controls; increased inflammatory cell infiltration was observed in the TCR-δ^−/−^mice after H5N1 virus infection (Figure 1C). The wet/dry weight ratio of the lung tissue, which is a measure of lung edema, was also markedly higher in the TCR-δ^−/−^mice 4 days after infection (Figure 1D). Taken together, these results demonstrate that a deficiency of γδ T cells results in a decreased survival rate and more severe lung injury following respiratory H5N1 infection, thus indicating that γδ T cells play a critical role in protection from H5N1 viral infection.

### HPAI H5N1 Virus Could Induce Human γδ T Cell Agglutination and Activation

**Figure 2:**
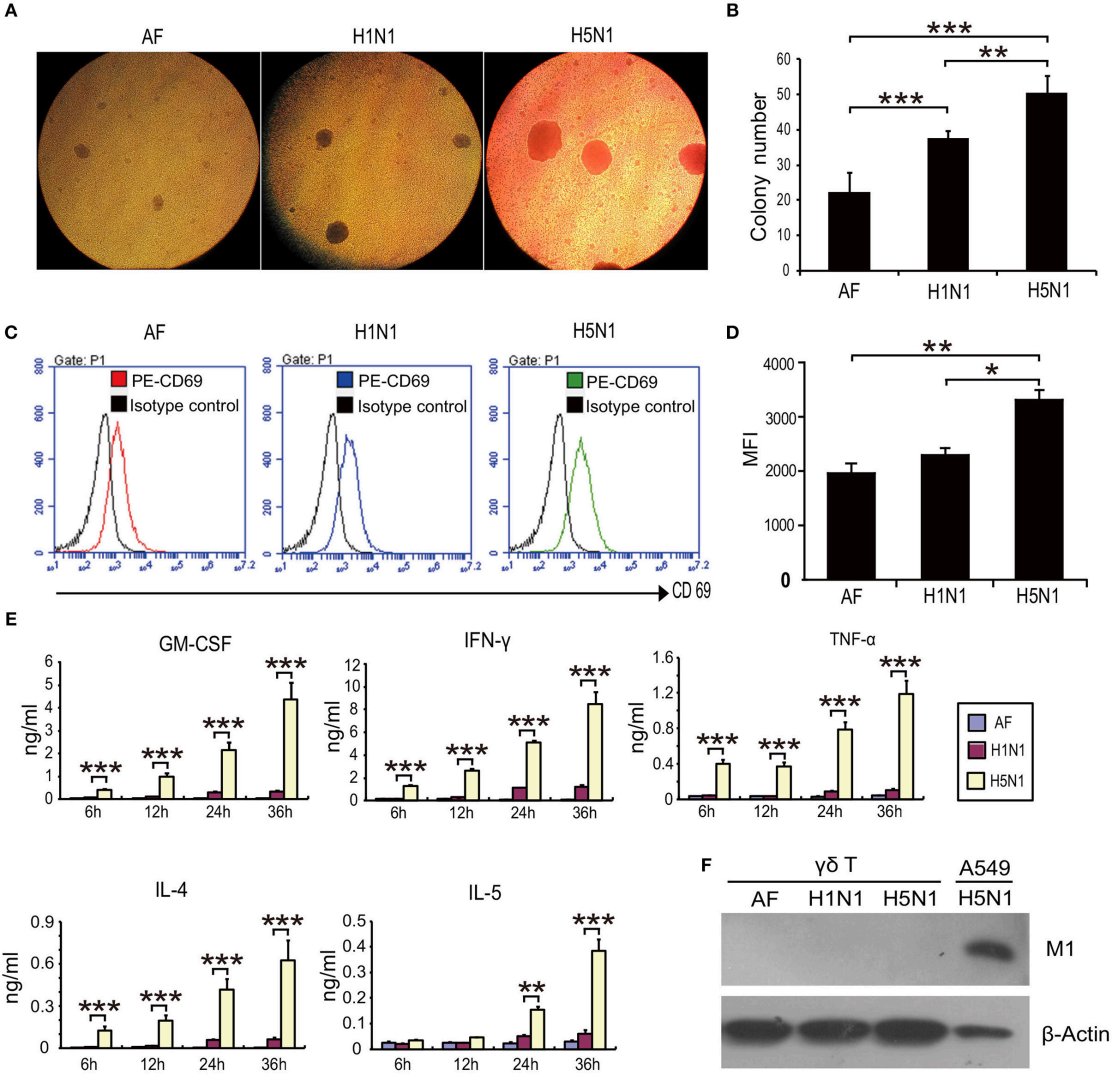
H5N1 virus induces the agglutination and activation of γδ T cells. γδ T cells were infected with HPAI H5N1 viruses or H1N1 viruses at an MOI of 4 for 12 h. **(A)** Representative images of agglutination of γδ T cells induced by viruses. Allantoic fluid (AF) with no viruses as control. **(B)** Quantification of the agglutination of γδ T cells induced by viruses. **(C)** Flow cytometry analysis of the CD69 expression on γδ T cells following viral infection. **(D)** Quantification of CD69 expression levels on γδ T cells from at least three independent experiments. **(E)** ELISA analysis of the cytokine secretion by γδ T cells following viral infection. **(F)** Detection of viral M1 protein by Western blotting following viral infection. Data are presented as the means ± SEMs. ^*^P < 0.05, ^**^P < 0.01 and ^***^P < 0.001, Student's *t-*test.

Next, we sought to explore the mechanism underlying γδ T cell-mediated protection against H5N1 viral infection. The interplay between HPAI H5N1 virus and human γδ T cells has not been reported. To determine whether HPAI H5N1 virus could directly induce human γδ T cell activation *in vitro*, human γδ T cells were isolated from healthy donors and expanded before infection with HPAI H5N1 virus at a multiplicity of infection (MOI) of 4 for 12 h. HPAI H5N1 virus induced severe agglutination of γδ T cells (Figures 2A,B). In addition, the expression of CD69 was significantly upregulated following HPAI H5N1 virus infection (Figures 2C,D). Furthermore, the secretion levels of the cytokines GM-CSF, IFN-γ, TNF-α, IL-4, and IL-5 in γδ T cells were markedly increased after HPAI H5N1 infection (Figure 2E). Nevertheless, the viral M1 protein could not be detected after HPAI H5N1 infection (Figure 2F). Taken together, these results indicate that γδ T cells could be activated by HPAI H5N1 virus but are resistant to H5N1 infection.

### Preparation and Purification of Trimeric and Monomeric rH5HA Proteins

**Figure 3:**
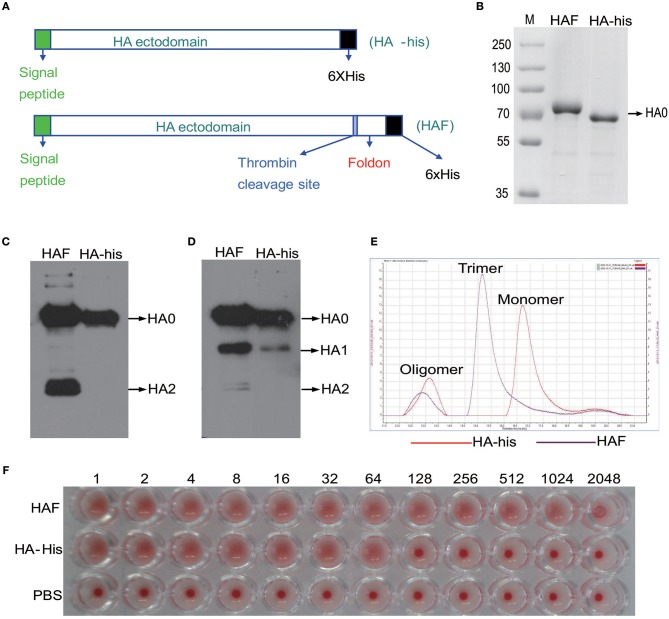
Preparation of HAF and HA-his proteins. **(A)** The scheme of vector design. HA-his vector was constructed to express HA-his monomers and HAF vector would express HA with a trimeric fold on sequence at the C-terminus to form HA trimers. These vectors were introduced into Sf9 cells with linearized baculovirus DNA as described in Materials and Methods to generate recombinant proteins that were purified in monomeric and trimeric forms and used in further studies. Purified rHA proteins were analyzed by Coomassie blue staining **(B)** and Western blotting with anti-His **(C)** and anti-HA **(D)** antibodies. **(E)** Purified HAF proteins existed as trimers and purified HA-his proteins existed as monomers, as determined by gel filtration chromatography; a few oligomeric proteins were present in both groups. **(F)** Hemagglutination activity analysis of purified rHA proteins.

Next we sought to determine how HPAI H5N1 viruses stimulate the activation of γδ T cells. HA is the most abundant antigen on the surface of influenza viruses. Therefore, we used recombinant hemagglutinin (rHA) proteins to investigate the interplay between the HPAI H5N1 virus and γδ T cells. Two forms of rHA proteins, namely HAF and HA-his, were constructed as previously reported (Figure 3A) (23). The expression of the rH5HA proteins was confirmed by Coomassie blue staining (Figure 3B) and by Western blotting using antibodies against the His tag (Figure 3C) and HA (Figure 3D). Gel filtration chromatography was further conducted to analyze the oligomerization of rHA proteins. The majority of the HAF protein appeared as an HA trimer, while the HA-his protein appeared as an HA monomer; a few oligomeric proteins were present in both groups (Figure 3E). Both the HAF and HA-his proteins showed hemagglutination activity (Figure 3F), indicating their ability to bind sialo-glycoconjugates on the erythrocytes. However, higher binding ability was observed for HAF proteins than for HA-his proteins (Figure 3F).

### HAF Proteins Induce γδ T Cell Agglutination and Activation

**Figure 4:**
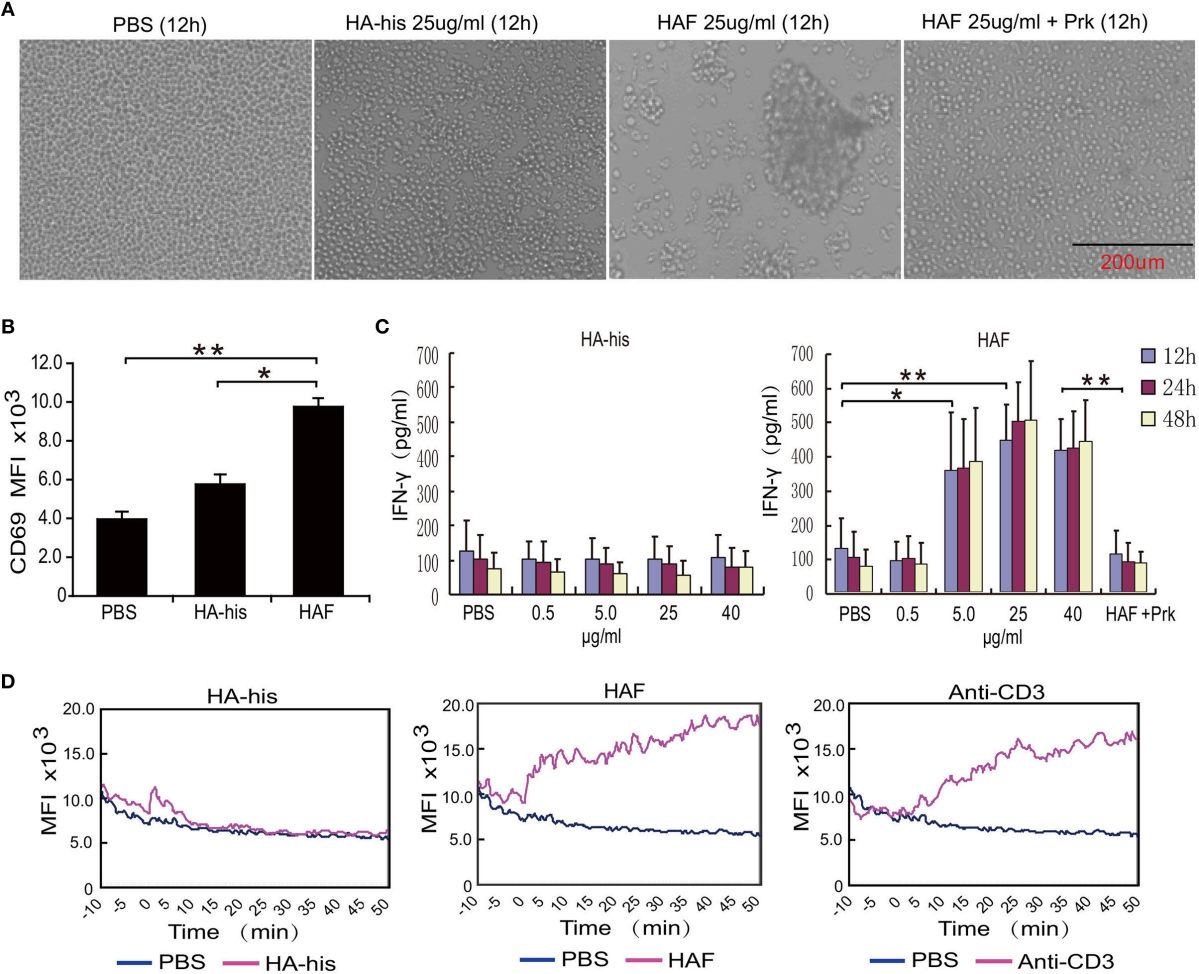
rH5HA proteins induce the agglutination and activation of γδ T cells. **(A)** Representative images of cultured γδ T cells which were seeded into 48-well plates and then treated with PBS, HAF or HA-his for 12 h. **(B)** Flow cytometry analysis of CD69 expression on γδ T cells following stimulation by HAF or HA-his proteins. **(C)** IFN-γ secretion by γδ T cells following stimulation by HAF or HA-his proteins. **(D)** Changes in the intracellular Ca^2+^ levels in γδ T cells following stimulation by HAF or HA-his proteins. Graphs in the panels represent the kinetics of the intracellular Ca^2+^ levels. Data are presented as the means ± SEMs. ^*^P < 0.05, ^**^P < 0.01, Student's *t-*test.

Consistent with the integration of the HPAI H5N1 virus, substantial agglutination of freshly isolated primary human γδ T cells was observed after incubation with HAF but not with HA-his proteins (Figure 4A). However, the agglutination of γδ T cells was not observed when HAF proteins were treated with proteinase K (Figure 4A). After γδ T cells were stimulated with HAF proteins, the expression level of CD69 on γδ T cells significantly increased. However, the expression level of CD69 increased slightly after γδ T cells were stimulated with HA-his proteins (Figure 4B). Furthermore, IFN-γ production markedly increased in a dose-dependent manner after γδ T cells were stimulated with HAF but not with HA-his proteins, and the stimulating effect of HAF proteins disappeared after proteinase K treatment (Figure 4C). As a key triggering signal, an increase in the intracellular free calcium concentration is rapidly induced upon γδ T cell activation (24). Consistent with anti-CD3 mAb treatment, delayed Ca^2+^ responses were observed in γδ T cells after incubation with HAF but not with HA-his proteins (Figure 4D). Taken together, these results suggest that rH5HA trimers but not the monomers could directly induce the agglutination and the activation γδ T cells.

### Sialic Acid Receptors Mediate HA Binding and γδ T Cell Activation

**Figure 5:**
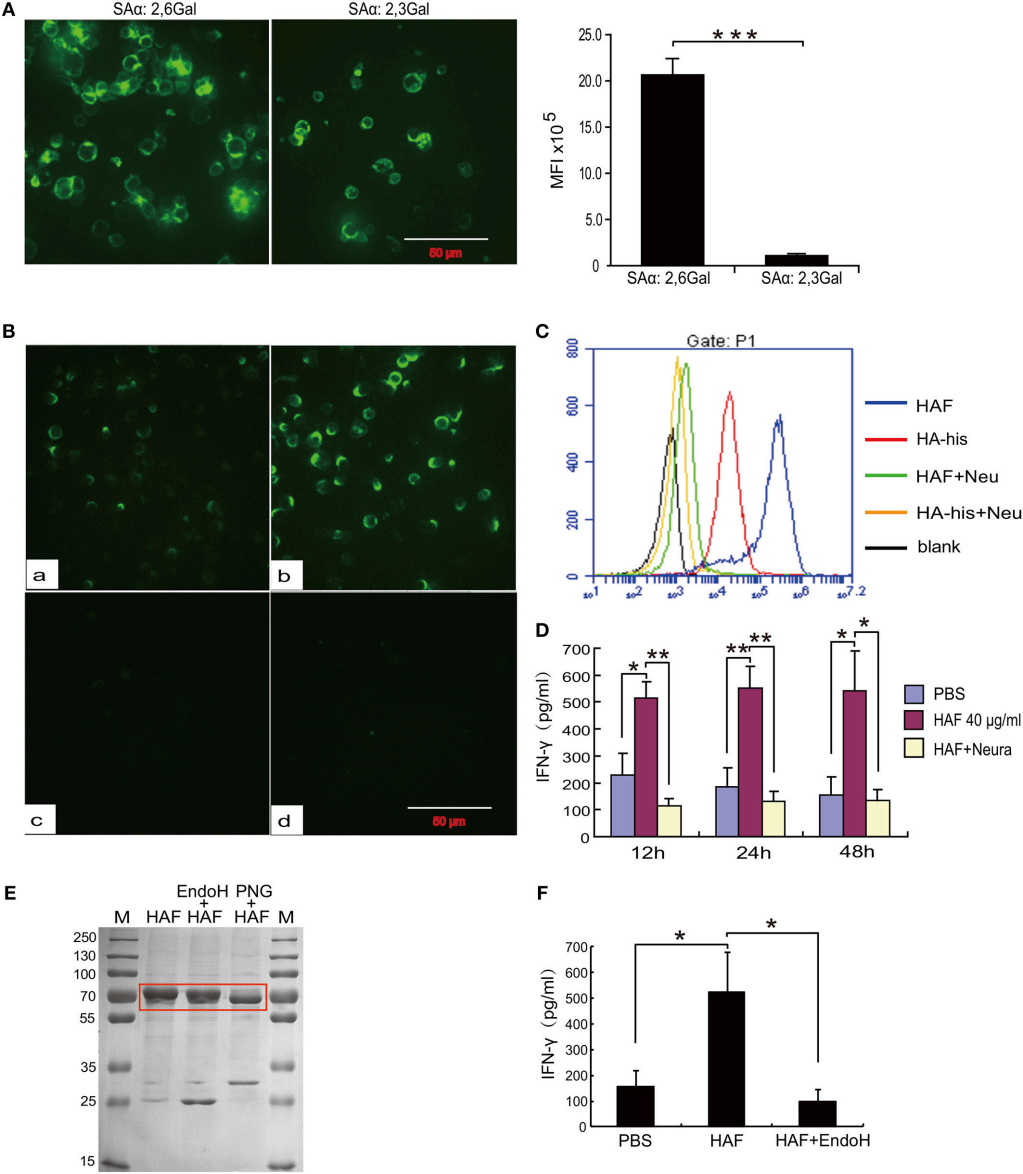
Sialic acid receptors mediate HA binding and γδ T cell activation. **(A)** Immunofluorescence detection of the expression levels of sialic acid receptors on the surface of γδ T cells. **(B,C)** Immunofluorescence detection of the binding activities of rHA proteins to γδ T cells before and after neuraminidase digestion. **(D)** ELISA detection of IFN-γ secretion of γδ T cells following neuraminidase digestion. **(E)** Coomassie blue staining of HAF after glycosidase treatment. **(F)** IFN-γ secretion by γδ T cells following Endo H treatment of rHA. Data are presented as the means ± SEMs (error bars). ^*^P < 0.05, ^**^P < 0.01 and ^***^P < 0.001, by Student's *t-*test.

To further determine the molecular mechanism underlying γδ T cell activation by rH5HA trimer stimulation, we first analyzed the expression of sialic acid receptors, which is a major determinant of the cellular tropism of the influenza virus, on the surface of γδ T cells. As previously reported, both α-2,3 and α-2,6 sialic acid receptors could be detected on γδ T cells (25). The expression level of α-2,6 sialic acid receptors was higher than that of α-2,3 sialic acid receptors (Figure 5A). In addition, both HAF and HA-his proteins bound directly to the surface of γδ T cells, and HAF proteins had the higher binding ability. However, neither HAF nor HA-his proteins could bind to the surface of γδ T cells after neuraminidase digestion (Figures 5B,C). Furthermore, the ability of HAF proteins to stimulate IFN-γ production disappeared after neuraminidase digestion (Figure 5D), indicating a requirement of sialic acid receptors for the HAF-induced γδ T cell activation. In addition, when HAF proteins were treated with endoglycosidase H (Endo H), only very little production of IFN-γ by γδ T cells could be detected (Figures 5E,F), suggesting that the glycosylation of HA also plays an important role in HAF-induced γδ T cell activation.

### Cyclosporin A Inhibits HA-Induced γδ T Cell Activation

**Figure 6:**
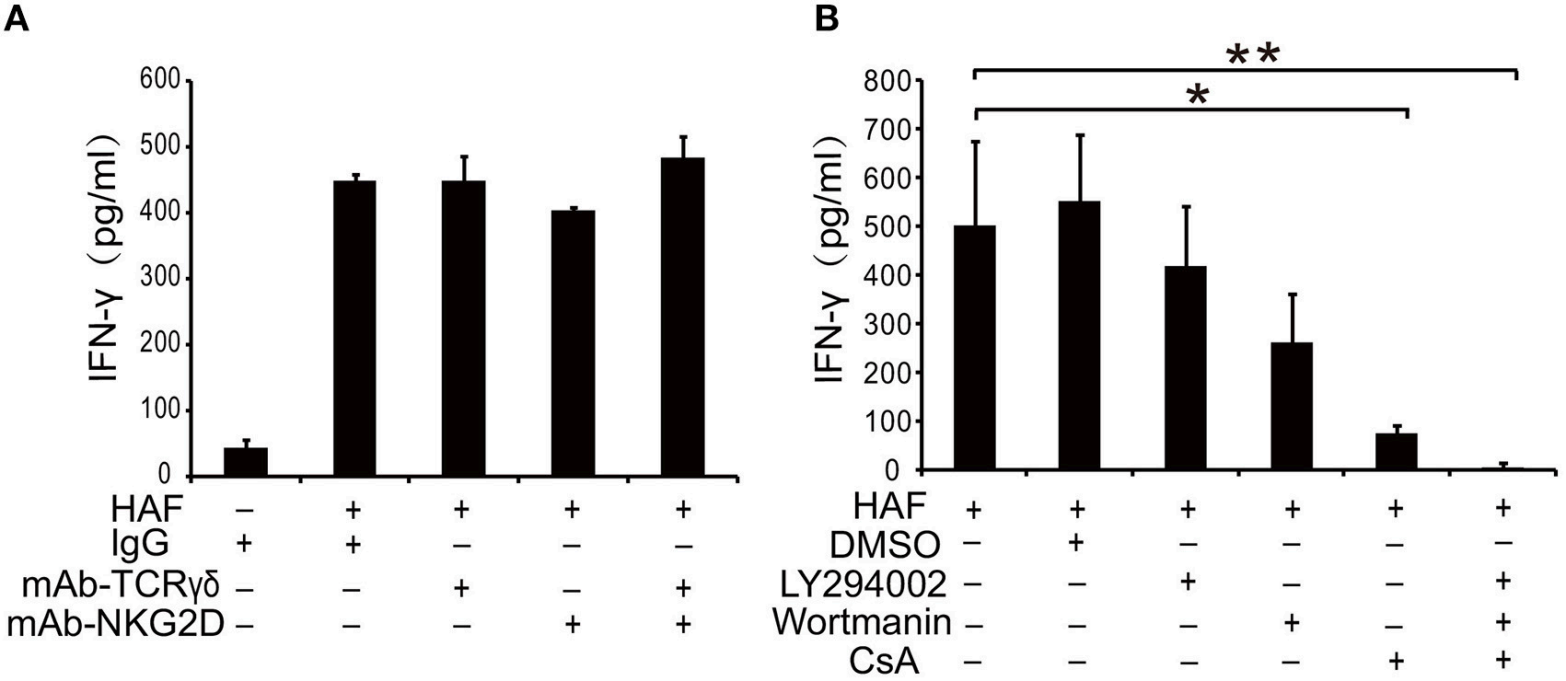
HA-induced γδ T cell activation could be blocked by the phosphatase calcineurin inhibitor CsA. **(A)** Inhibition assay of γδ T cells with TCR γδ and NKG2D mAbs. **(B)** Effect of pharmacological inhibitors on HA-induced γδ T cell activation. Data are presented as the means ± SEMs. ^*^P < 0.05 and ^**^P < 0.01, by one-way analysis or variance (ANOVA) with Tukey's *post hoc* test.

TCR γδ and NKG2D are two most important receptors on the surface of γδ T cells to initiate γδ T cell-mediated immune response (26, 27). However, the treatment of γδ T cells with TCR γδ and NKG2D mAbs could not block HAF-induced γδ T cell activation (Figure 6A). Then we applied several pharmacological inhibitors to explore the signal transduction pathways involved in HA-induced γδ T cell activation. HA-induced γδ T cell activation could be inhibited by the phosphatase calcineurin inhibitor CsA but not by the phosphatidylinositol 3-kinase (PI3-K) inhibitors wortmannin and LY294002 (Figure 6B). These results reveal that calcineurin-nuclear factor of activated T cell cytoplasmic (NFATc) pathway plays a critical role in HA-induced γδ T cell activation.

## Discussion

Although the antibacterial and tumoricidal roles of γδ T cells have been well studied, their roles during influenza virus infection remain poorly understood. In this study, we demonstrate for the first time that γδ T cells could be directly activated by HPAI H5N1 viruses and that γδ T cells play an important role in the defense against HPAI H5N1 infection. The trimers but not monomers of HPAI H5N1 viral HA proteins could also directly activate γδ T cells, and this activation depends on sialic acid receptors on the surface of γδ T cells and on the glycosylation of HA proteins. Moreover, HA-induced γδ T cell activation could be inhibited by the phosphatase calcineurin inhibitor CsA. These findings provide a further understanding the mechanism underlying γδ T cell-mediated innate and adoptive immune responses against HPAI H5N1 viral infection.

Unlike αβ T cells, γδ T cells constitute only a small proportion (1–10%) of the T lymphocytes in the peripheral blood and organs. One of the most important features of γδ T cells is their tropism for epithelial surfaces, including those of the skin and liver as well as the mucosae of respiratory, reproductive and digestive organs; γδ T cells migrate to these surfaces during their early development and persist there as resident cells (28). In addition, γδ T cells acquire a preactivated phenotype that allows the rapid induction of effector functions following the detection of cellular stress and infection. Previous reports described the role of γδ T cells in influenza infection in a mouse model. The number of γδ T cells resident in the lung was increased after influenza A virus (H3N2) infection (10). Dr. Mozammal Hoq et al further reported that after γδ T cells depletion *in vivo*, the resistance of mice to influenza A virus (H3N2 and H1N1) infection was abrogated (11). Recent studies demonstrated that IPP-expanded γδ T cells originating from PBMCs could kill monocyte-derived macrophages infected with human (H1N1) and avian (H9N2 or H5N1) influenza viruses (15, 16). γδ T cells in PBMCs could be rapidly activated by influenza virus (H1N1, H2N2, and H3N2) infection via a mechanism related to the mevalonate pathway (17). However, the *in vivo* role of γδ T cells in HPAI H5N1 infection and the direct effects of HPAI H5N1 viruses on human γδ T cells have not yet been reported.

In this study, we demonstrate for the first time that γδ T cell deficiency results in a decreased survival rate and more severe lung injury following respiratory H5N1 infection, which indicates that γδ T cells play a critical role in the infection process. However, Xue et al. recently reported that the depletion of γδ T cells, especially the Vγ4+ γδ T cell subset, significantly rescued the weight loss induced by H1N1 infection and improved the survival rate by reducing IL-17A secretion and immunopathological injury (29). These contradictory results are likely due to the use of different influenza virus strains and treatment methods. In our study, the mice were infected with the HPAI H5N1 virus, which likely induces more severe lung injury than does the H1N1 virus. Moreover, our animal experiments were conducted in TCR-δ^−/−^ mice, which completely lack γδ T cells; however, γδ T cells may not have been completely depleted by the anti-mouse TCR γδ mAbs used in the later study.

γδ T cells in PBMCs could be activated by influenza virus (H1N1, H2N2, and H3N2) infection (17); however, the interplay between the HPAI H5N1 virus and γδ T cells has not been reported. In our study, we demonstrate for the first time that the HPAI H5N1 virus could directly induce severe γδ T cell agglutination and activation *in vitro*. The expression of CD69 and the secretion levels of the cytokines GM-CSF, IFN-γ, TNF-α, IL-4, and IL-5 in γδ T cells were markedly increased after HPAI H5N1 infection. Therefore, γδ T cells could participate in viral clearance directly by the production of IFN-γ and indirectly through the regulation of other immune effector cells by the production of immunomodulatory cytokines. Moreover, the ability of the HPAI H5N1 virus to activate γδ T cells was higher than that of H1N1; this result is consistent with the *in vivo* virulence of these strains. H5N1 has been reported to infect many types of immune cells, including dendritic cells (DCs), monocytes, macrophages, neutrophils, natural killer (NK) cells, and B cells (30–33). Nevertheless, our study confirms that γδ T cells are resistant to HPAI H5N1 infection, consistent with previous reports showing that γδ T cells were not susceptible to influenza A virus infection (34, 35). Therefore, we speculate that the rapid activation response and ability to resist HPAI H5N1 infection increase the advantage of γδ T cells in controlling viral infection compared with that of other immune cells.

As the most abundant antigen on the surface of the influenza virus, HA is responsible for host receptor binding, internalization, and the subsequent membrane fusion events in the infected cell (23, 36). In its mature form, natural HA is a homotrimer with multiple glycosylation sites. In our study, two forms of recombinant hemagglutinin, namely, HA trimers and HA monomers, were successfully expressed and purified. Consistent with previous reports, HAF proteins could form HA trimers like the natural form of HA proteins and had a fold on sequence at the C-terminus, and HA-his proteins existed mainly as HA monomers (23, 36). Although a few oligomeric proteins were present in both groups, we believe that the oligomers in the HAF group were constructed with HA trimers, while those in the HA-his group were HA monomers. Both the HAF and HA-his proteins showed hemagglutination activity; however, a higher binding ability was observed for HAF than that of HA-his, which may be related to the protein conformation. Furthermore, Chih-Jen et al. found that the immunogenicity of HA trimers was stronger than that of HA monomers in mice immunized with these recombinant HA proteins (37). In our previous study, we found that rHA proteins derived from HPAI H5N1 could activate γδ T cells in PBMCs (25). Here, we further confirmed that HAF proteins could not only induce γδ T cell agglutination but could also directly activate γδ T cells via mechanisms such as CD69 upregulation, IFN-γ secretion and increased intracellular calcium mobilization.

In our previous study, we found that both α-2,3 and α-2,6 sialic acid receptors could be detected on γδ T cells (25). Here, we further confirmed that the expression level of α-2,6 sialic acid receptors was higher than that of α-2,3 sialic acid receptors; this result is consistent with a previous report that α-2,6 sialic acid is dominant on epithelial cells in the human nasal mucosa (38). Both the HAF and HA-his proteins bound directly to the surface of γδ T cells, and HAF proteins were observed to have the higher binding ability, consistent with the agglutination ability of their γδ T cells. Furthermore, we confirmed that the activation of γδ T cells induced by HA trimers was dependent on sialic acid receptors; HA trimers could not bind to the surface of γδ T cells, and the stimulation of IFN-γ production by HA trimers disappeared after neuraminidase digestion. HA from human-derived viruses preferentially recognizes α-2,6 sialic acid receptors, whereas HA from avian viruses preferentially recognizes α-2,3 sialic acid receptors (38). Our results confirm that although the expression level of α-2,3 sialic acid receptors is lower than that of α-2,6 sialic acid receptors, the expression is sufficient to induce γδ T cell activation. Glycosylation plays a critical role in HA cleavage, structural stability and pathogenicity in HPAI H5N1 virus infection (39). In our study, we found that HA glycosylation plays an important role in γδ T cell activation; the production of IFN-γ by γδ T cells was nearly absent after the treatment of HAF proteins with Endo H.

Sialic acid receptors are usually attached to the terminal galactose of cell surface glycoproteins by α-2,3 or α-2,6 linkages. TCR γδ and NKG2D, which play critical roles in antigen recognition and γδ T cell activation, are the two most important glycoprotein receptors on the surface of γδ T cells. We initially examined the relative contribution of TCR γδ and NKG2D in rHA-induced γδ T cell activation. However, in our study, treating γδ T cells with anti-TCR γδ and anti-NKG2D mAbs could not inhibit HA trimer-induced γδ T cell activation. Nevertheless, our result cannot totally rule out the participation of TCR γδ and NKG2D in HA-induced activation, because the mAbs may not completely block the HA binding sites. Therefore, the specific glycoprotein receptors mediating the binding of HA to γδ T cells need further study. To determine the signaling pathways involved in the HA trimer-induced activation of γδ T cells, we treated HA trimer-stimulated γδ T cells with the PI3-K inhibitors wortmannin or Ly294002 and with the immunosuppressive drug CsA. We found that HA trimer-induced γδ T cell activation was inhibited by CsA but not by wortmannin or LY294002. CsA is a phosphatase calcineurin inhibitor that affects a subset of calcium-associated signaling events similar to those involved in T cell activation (40). The inhibitory effect of CsA was also consistent with our discovery that HA trimers could induce an increase in the intracellular free calcium concentration upon γδ T cell activation.

In summary, our study demonstrates for the first time that γδ T cells play an important role in the defense against HPAI H5N1 virus infection. γδ T cells could be directly activated by HPAI H5N1 viruses through natural trimers of HA proteins; this ability was dependent on sialic acid receptors on the surface of γδ T cells and on the HA glycosylation. Furthermore, HA-induced γδ T cell activation can be inhibited by CsA but not by wortmannin or LY294002. Our results provide new insights about the immunoprotective role of γδ T cells in the defense against HPAI H5N1 virus infection and provide a novel strategy for controlling influenza virus infection in the future.

## Ethics Statement

This study was carried out in accordance with the recommendations of the animal facility of the Institute of Basic Medical Sciences, Peking Union Medical College and the Institute of Military Veterinary Medicine, Academy of Military Medical Sciences, in accordance with governmental and institutional guidelines. The protocol was approved by the committee of Institute of Basic Medical Sciences, Peking Union Medical College.

## Author Contributions

PD, XJ, and YY contributed to the experiments on mice. PD, XJ, and MC performed all the *in vitro* experiments. SZ, HW, and MC contributed to mouse husbandry. PD and XJ performed the statistics. HC, YH, and LC analyzed the data and provided key ideas. PD, JZ, and WH designed the experiments as well as wrote the paper.

### Conflict of Interest Statement

The authors declare that the research was conducted in the absence of any commercial or financial relationships that could be construed as a potential conflict of interest.
